# Escape into Social Media? A 4-Week Tracking Study on Nomophobia and Smartphone Coping

**DOI:** 10.3390/healthcare14091125

**Published:** 2026-04-22

**Authors:** Jiahao Li, Yang Chu, Shan Liu, Yanfang Liu, Jie Xu

**Affiliations:** 1 Center for Psychological Sciences, Zhejiang University, Hangzhou 310058, China; jiahao_li@zju.edu.cn (J.L.); yangchu@zju.edu.cn (Y.C.); 13235966023@163.com (S.L.); 2 Huawei Technologies Co., Ltd., Beijing 100080, China; yanfang.liu@aliyun.com; 3 Department of Psychology, Lingnan University, Hong Kong SAR, China; 4 Lingnan University Cognitive Science Research Centre, Lingnan University, Hong Kong SAR, China

**Keywords:** nomophobia, problematic smartphone use, I-PACE model, longitudinal study, social media use

## Abstract

**Highlights:**

**What are the main findings?**

- Objective tracking reveals that nomophobia is associated with increased duration and frequency of social media use.
- Smartphone use frequency among high-nomophobia individuals escalates specifically when negative emotional states coincide with periods of low situational busyness.

**What are the implications of the main findings?**

- Advances the transition in problematic smartphone use research from a “total usage” metric to a “context-sensitive” approach by integrating objective behavioral tracking with intensive longitudinal data.
- Identifies nomophobia as a predisposing factor that moderates situational reactivity, amplifying smartphone coping when negative affect coincides with low busyness.

**Abstract:**

**Background**: Nomophobia, the fear of being without a mobile phone, has become an increasing public health concern. While existing theories suggest that smartphones often serve as tools for emotional regulation, the situational mechanisms driving these compensatory behaviors remain under-explored. This study investigated how nomophobia levels interact with daily emotional fluctuations and busyness to influence smartphone-based coping patterns. **Methods**: We employed an intensive longitudinal approach combining objective smartphone tracking with a 4-week daily diary design. Thirty-seven participants were monitored, yielding 837 daily observations. Smartphone use was categorized into Instant Messaging (IM), Social Media Use (SMU), and Non-social Use (NSU). Multilevel linear regression analyzed the interaction effects on usage metrics. **Results**: Nomophobia significantly correlated with the duration and frequency of SMU, but not IM or NSU. A significant three-way interaction was observed: individuals with high levels of nomophobia exhibited a significantly increased frequency of overall usage, SMU and NSU when experiencing negative emotions during periods of low busyness. In contrast, low-nomophobia individuals maintained stable usage patterns regardless of situational stressors. **Conclusions**: By conceptualizing smartphone usage as a behavioral proxy for the coping process, this study provides preliminary evidence that nomophobia is associated with a situation-dependent coping pattern, primarily involving increased social media usage. These findings underscore the importance of integrating situational contexts and underlying coping processes to better understand and manage problematic smartphone use.

## 1. Introduction

Smartphones have become a pervasive infrastructure of modern life [1]. Beyond being tools for communication and productivity [2,3], smartphones often act as a “security blanket” and a “first-aid-in-the-pocket”, helping individuals manage daily situations and regulate emotions [4,5]. However, this constant connectivity has introduced significant public health concerns [6], most notably nomophobia—the “no mobile phone phobia” [7]. Nomophobia is conceptualized as the situational anxiety or discomfort experienced when individuals are disconnected from their digital devices [8,9]. In addition to fear-like symptoms, it shares key features with behavioral addictions, including compulsivity and functional impairment [10]. Researchers have proposed its inclusion in the DSM framework to facilitate a more profound investigation into its underlying pathology [8,10,11].

Current research on nomophobia has primarily focused on its prevalence across diverse demographics and its associations with various psychological comorbidities [12]. However, the specific behavioral patterns and psychological mechanisms driving this phenomenon remain poorly understood. A major limitation is the field’s reliance on static, cross-sectional self-reports [13], which fail to capture the dynamic, situational nature of smartphone dependency in daily life. To address these gaps, the present study aligns with the Interaction of Person-Affect-Cognition-Execution (I-PACE) model, utilizing objective smartphone tracking coupled with a 4-week daily diary approach. We pursue two primary objectives: (a) an exploratory aim to delineate the behavioral signatures of nomophobia; and (b) a confirmatory aim to elucidate the underlying psychological mechanisms by examining how nomophobia moderates the intensity of smartphone behavioral reactivity to emotional and situational demands.

### 1.1. Nomophobia and Behavioral Smartphone Use

Smartphone use is multifaceted, involving various patterns and motivations [14]. Duration and frequency are commonly employed as primary indicators. Prior studies generally report a correlation between these metrics and symptoms of nomophobia [11,15]. However, these studies have largely relied on self-reported methods [16,17], which are susceptible to recall biases and social desirability effects [2,18]. In response, researchers have turned to smartphone-logged data for a more accurate and valid approach. These studies confirm that nomophobia correlates with both duration and frequency, but which indicator is more critical remains unclear [19,20]. To resolve this ambiguity, our study uses objective smartphone tracking to simultaneously assess both duration and frequency.

Beyond overall usage, the type of smartphone activity may also play a key role in nomophobia. Prior work often distinguishes between social and non-social use, but findings are mixed [21,22]. For instance, Jeong et al. [23] discovered that the use of social networking services (SNSs) was a more significant indicator of problematic smartphone use, whereas Casale et al. [24] found that social use does not necessarily predict nomophobia. One explanation for these inconsistencies is that many studies fail to differentiate between active and passive social use [25]. Active social use, characterized by direct communication such as messaging and calling, fosters interpersonal relationships [26]. Passive social use involves content consumption without direct communication (e.g., scrolling through news feeds and viewing posts) [26]. Evidence suggests that passive social use is more strongly linked to problematic smartphone use [27] and reduced psychological well-being [26,28].

Drawing on prior research [29,30,31], we classify smartphone use into three categories based on primary app functionality. Instant Messaging (IM) serves for communication (e.g., Messenger, WhatsApp). Social Media Use (SMU) typically involves passive content consumption (e.g., Facebook, Twitter, TikTok). Non-Social Use (NSU) encompasses all applications outside these two social use categories. IM involves unpredictable and potentially stressful social interactions [32,33], whereas SMU provides immediate gratification and a “micro escape” [30,34] to alleviate anxiety. Given these divergent psychological profiles, we suggest that these usage categories may exhibit differential sensitivity in reflecting the severity of nomophobia. Consequently, we propose the following exploratory research question:

**Research Question:** How do the duration/frequency of IM, SMU, and NSU relate to the level of nomophobia?

### 1.2. Nomophobia in Smartphone-Based Coping

Beyond examining static usage patterns, it is crucial to understand why and when individuals with nomophobia turn to their smartphones. The I-PACE model provides a theoretical framework for the process underlying the development and maintenance of Internet use disorders [35,36]. Recent studies have drawn upon this model to understand nomophobia and smartphone use [34,37,38]. According to the I-PACE model, specific smartphone use emerges from interactions among predisposing factors, affective and cognitive responses, and executive functioning. With the involvement of conditioning processes, these associative interactions are gradually reinforced and tend to become automated.

Within this model, negative emotional states (e.g., stress, anxiety, depression, or boredom) serve as the core internal stimuli that activate an individual’s response system. The corresponding cognitive and behavioral efforts deployed to manage these stressful person-environment transactions and their associated emotions are conceptualized as “coping” [39]. Due to their high accessibility and immersive nature, smartphones have naturally become a preferred coping tool for modern individuals. Smartphone coping specifically refers to the use of smartphones as a tool to manage stress or negative emotions, including attention diversion, problem-solving, and seeking emotional support [2,40]. Consequently, the dynamic interplay between emotional states and objective smartphone use can be analyzed to reflect the underlying coping process. However, empirical evidence regarding the relationship between emotional states and smartphone use presents a complex and inconsistent picture [41,42,43]. This inconsistency suggests that individual differences and contextual constraints must be carefully considered when investigating emotion-induced smartphone use.

Under the I-PACE framework, nomophobia acts as a crucial predisposing factor that influences the smartphone-based coping process. Nomophobia is typically accompanied by higher trait anxiety [44] and a higher intolerance of uncertainty [45], with affected individuals commonly experiencing difficulties in emotion regulation [6]. Lacking intrinsic emotion regulation capabilities [46], individuals with high levels of nomophobia often view their smartphones as an external reliance for reassurance seeking or distraction [47,48]. Furthermore, the persistent anxious state associated with nomophobia consumes central executive resources, thereby increasing distractibility, reducing working memory capacity, and weakening overall executive functioning [49]. Consequently, nomophobia may significantly impair an individual’s inhibitory control when exposed to smartphone-related cues. As a result, these individuals tend to develop rigid coping styles [50], wherein smartphone use becomes their primary mechanism for emotional regulation. Accordingly, when faced with emotional distress, individuals with nomophobia are highly likely to exhibit a surge in smartphone use.

Furthermore, smartphone use is influenced not only by emotional states and individual coping styles, but also by contextual factors such as busyness. The theory of limited resources suggests that attention, as a limited resource, is allocated to various demands [51]. When individuals are busier, they tend to use smartphones less, as their attention is occupied with tasks at hand. Conversely, during less busy periods, or when experiencing underload, individuals may increase smartphone use to combat boredom [52]. We propose that the level of nomophobia will moderate how emotion and busyness influence smartphone use. Our hypothesis is as follows:

**Hypothesis** **1.** *Individuals with higher levels of nomophobia are more likely to increase smartphone use when facing negative emotions, particularly on less busy days.*

## 2. Methods

### 2.1. Participants and Procedure

A convenience sample of 40 participants was recruited from East China via online advertisements. Three participants withdrew during the tracking period, resulting in a final effective sample of 37 participants (Ages 18–39, *M* = 26, *SD* = 5.02; 48.6% female). All participants provided informed consent and received monetary compensation. The protocol was approved by the Institutional Research Ethics Board. To protect participant privacy, a de-identification protocol was implemented; no sensitive personal content (e.g., messages, call logs) was collected.

Prior to the study, participants’ nomophobia levels and demographic information were collected. Smartphone use was tracked using a custom-developed Android application. The application automatically logged the app name, start time, and duration of each usage session. Concurrently, participants completed daily assessments of their overall emotional states and perceived busyness via an online survey at 10:00 PM for approximately 4 weeks. After excluding missing entries, 837 valid person-day observations were retained for analysis (*M* = 22.6 days per participant).

### 2.2. Measures

#### 2.2.1. Self-Report Measures

Daily Survey: Daily emotional valence and perceived busyness were assessed using single-item 5-point scales. Single-item assessments were employed to optimize compliance and reduce burden. Evidence indicates such measures maintain robust concurrent and predictive validity in intensive longitudinal designs [53]. Emotional valence ranged from 1 (Very Negative) to 5 (Very Positive); busyness ranged from 1 (Very Relaxed) to 5 (Very Busy).

Nomophobia Questionnaire (NMP-Q): The nomophobia levels were assessed using the Chinese NMP-Q [54], adapted from the original version [55]. The NMP-Q includes 18 items covering four dimensions of nomophobia: not being able to communicate, losing connectedness, not being able to access information, and giving up convenience. Each item was rated on a 7-point Likert scale, from “1—Strongly disagree” to “7—Strongly agree”, with higher scores indicating a higher severity of nomophobia. The questionnaire demonstrated excellent internal consistency with a Cronbach’s alpha of 0.95. Participants were divided into two groups based on their nomophobia levels following the previous research [56]. The classification involved converting raw scores into Z-scores within the sample, where scores below the mean indicated “Low Nomophobia”, signifying an “absence or low level of nomophobia”, while scores above the mean were marked as “High Nomophobia”, including either “mild or severe level of nomophobia” [54,57].

Demographics: We collected relevant socio-demographic characteristics, including gender, age, occupation, and other relevant details. Additionally, we administered personality-related questionnaires, though they were not included in the analysis.

#### 2.2.2. Smartphone Use Measures

We measured overall app use (ALL) and classified smartphone applications into three categories: IM, SMU, and NSU. This classification is based on iiMedia-Research [58] standards, which were also referenced in previous studies such as Lin et al. [59]. Specifically, the IM category incorporates widely used apps such as WeChat (dubbed as “WhatsApp of China”) and QQ. The SMU category contains popular social media apps such as Weibo (akin to Twitter) and TikTok. The NSU category is a broader classification, encompassing all apps that are not included in either the IM or SMU categories.

We utilized two behavioral metrics. “Duration” tracks the daily total minutes spent on each app category. “Frequency” counts the daily number of app sessions within a category. Employing these metrics, we derived eight distinct measures: D-ALL (duration for ALL), F-ALL (frequency for ALL), D-IM (duration for IM apps), F-IM (frequency for IM apps), D-SMU (duration for SMU apps), F-SMU (frequency for SMU apps), D-NSU (duration for NSU apps), and F-NSU (frequency for NSU apps).

### 2.3. Data Analysis

First, descriptive statistics and correlation analyses were conducted. Next, to address our research question and hypothesis, we structured our analysis into two distinct phases, both categorizing nomophobia into high and low levels. Phase 1: Independent t-tests examined between-person (Level-2) differences in average usage. These tests were treated as exploratory preliminary analyses. Phase 2: Multilevel modeling was employed to test the cross-level three-way interaction (nomophobia × emotional valence × busyness). Level-1 predictors (emotional valence, busyness) were group-mean centered [60]. Given our Level-2 sample size (*N* = 37), participants were modeled as random intercepts, but slopes were fixed to ensure model stability and convergence [61]. Gender and age were included as covariates, with weekday/weekend and the sequence of days as controlled temporal factors.

Finally, we conducted sensitivity analyses. For the between-person t-tests, calculations indicated that achieving a target power of 0.80 (α = 0.05) with our Level-2 sample size requires a minimum detectable effect size (MDES) of approximately 0.94, acknowledging that this preliminary step is inherently underpowered for smaller effects. For the multilevel models, we conducted a Monte Carlo simulation (1000 iterations) [62]. The simulation parameters were grounded in our empirical data structure, specifically incorporating the valid days per participant and observed intra-class correlation (ICC ≈ 0.60). Assuming an α level of 0.05, a target power of 0.80, the simulation revealed an MDES of 0.20 for cross-level interactions, corresponding to a small-to-medium effect.

All statistical analyses were executed in the R software (Version 4.2.2) [63].

## 3. Results

### 3.1. Descriptive Statistics and Correlations

Table 1 presents the descriptive statistics and the correlations for our measures. We found that participants used their smartphones on average for 313 min per day, closely aligning with previously reported figures [64,65,66]. Across all use categories, we noted a strong correlation (0.74 to 0.84) between duration and frequency of smartphone use. Meanwhile, the average emotional states of participants showed no significant correlation with the nomophobia scale. Notably, we discovered a significant positive correlation between the nomophobia scale and both the average daily duration and frequency of SMU throughout the data collection period. In contrast, nomophobia showed no significant correlation with other smartphone use metrics.

### 3.2. Nomophobia and Smartphone Use

**Figure 1:**
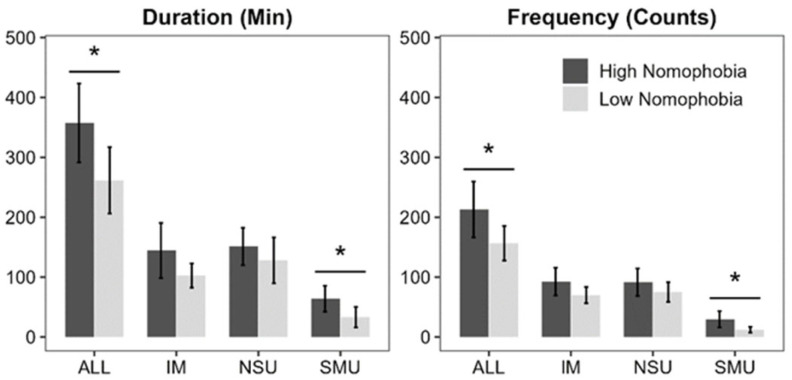
Comparison of smartphone use metrics between high and low nomophobia groups across specific application categories. The left panel illustrates the daily use duration (minutes), while the right panel shows the frequency of use (counts). Error bars represent 95% confidence interval. Asterisks (*) denote statistically significant differences between the two nomophobia groups at *p* < 0.05. *N* = 37.

**ALL:** Analysis showed that individuals in the high nomophobia group used their smartphones more frequently and for longer periods than those in the low nomophobia group. Significant differences were found in both use duration (*t*(35) = 2.20, *p* = 0.04) and frequency (*t*(35) = 2.04, *p* = 0.05), indicating a direct relationship between nomophobia severity and increased smartphone interaction (Figure 1).

**IM:** The analysis revealed that the differences in IM duration (*t*(35) = 1.66, *p* = 0.11) and frequency (*t*(35) = 1.67, *p* = 0.10) over the data collection period were not significant, suggesting that the level of nomophobia does not impact how often or how long individuals use instant messaging.

**SMU:** We identified a significant positive correlation between nomophobia levels and social media use. Individuals with higher levels of nomophobia exhibited longer durations (*t*(35) = 2.21, *p* = 0.04) and higher frequencies (*t*(35) = 2.40, *p* = 0.02) of social media use. This finding emphasizes the role of nomophobia in driving more extensive social media engagement.

**NSU:** NSU showed no significant association with levels of nomophobia. There was no significant variation in the duration (*t*(35) = 0.91, *p* = 0.37) or frequency (*t*(35) = 1.15, *p* = 0.26) of NSU activities based on nomophobia, indicating that such smartphone use is not influenced by nomophobia.

### 3.3. Interaction Effects of Nomophobia on Smartphone Use

**Figure 2:**
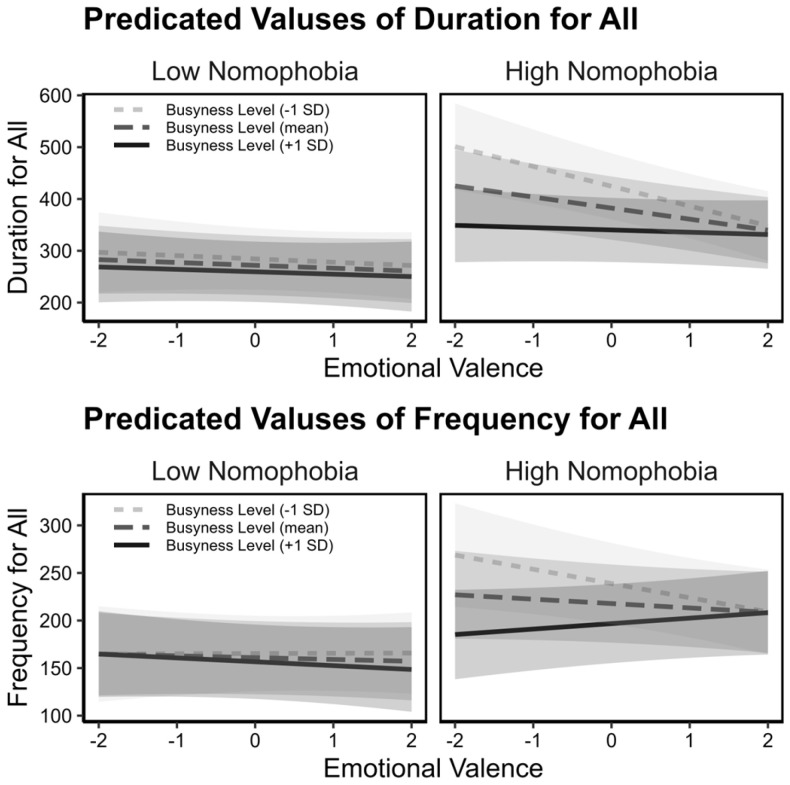
Marginal effect plots illustrating the 3-way interaction (Emotional Valence × Busynesse × Nomophobia Level) on overall smartphone use. The upper panels display predicted values for usage duration, and the lower panels show usage frequency. The interaction patterns reveal that while individuals with low nomophobia maintain stable usage regardless of situational changes, those with high nomophobia significantly increase their smartphone engagement when experiencing negative emotional states during periods of low busyness. The shaded area around each line represents a 95% confidence interval. Observations = 837, *N* = 37.

Our multilevel regression analysis was conducted to examine how nomophobia levels moderate smartphone-based coping in response to daily emotional fluctuations and situational busyness. The detailed outcomes of these models are presented in Table 2. Our hypothesis posited that individuals with higher levels of nomophobia would increase smartphone use during negative emotional states, particularly in less busy periods, which led us to examine the intricate three-way interaction between emotional valence, busyness, and nomophobia levels. Due to consistent patterns observed in both the frequency and duration across various smartphone use behaviors, we focused our report on overall smartphone use, as illustrated in Figure 2.

The graphs in Figure 2 illustrate that individuals with low nomophobia exhibit minimal changes in smartphone use with varying emotional valence and busyness, as indicated by the stable trend lines. This indicates a uniform use pattern irrespective of their emotional state or level of busyness. In contrast, high nomophobia individuals show a different pattern: their smartphone use diminishes as busyness increases; they engage with their smartphones consistently during times of high busyness, unaffected by emotions. Yet, notably, as busyness decreases, those with high nomophobia tend to increase their smartphone use if they experience a more negative emotion. The significant three-way interaction effect on the frequency of overall smartphone use (*B* = 9.87, β = 0.11, *p* = 0.02) supports this finding. Although the trend for overall smartphone use duration is similar, the interaction is not significant (*B* = 12.9, β = 0.09, *p* = 0.07).

**IM:** The three-way interaction of nomophobia level, busyness, and emotional valence had no significant impact on either the duration (*B* = −1.16, β = −0.01, *p* = 0.78) or the frequency (*B* = 1.45, β = 0.03, *p* = 0.55) of IM. However, a clear trend emerged when focusing on the impact of emotional valence alone: IM declined as individuals’ emotional states became more positive. This pattern was particularly pronounced in the duration of IM (*B* = −8.76, β = −0.09, *p* < 0.01) and to a lesser extent for the frequency of IM (*B* = −2.01, β = −0.04, *p* = 0.24).

**SMU:** The pattern of SMU aligns with the observed overall smartphone use. Specifically, we noted that individuals with high levels of nomophobia tend to increase the frequency and duration of social media use as emotional states become more negative, especially when individuals are less busy. Conversely, individuals with lower levels of nomophobia exhibit less variation in their social media use in response to changes in emotional valence and busyness, indicating a more stable pattern of engagement. Statistical analyses provide robust support for these observations. Significant three-way interaction effects were noted for the duration of SMU (*B* = 7.07, β = 0.14, *p* = 0.02) and the frequency of SMU (*B* = 2.31, β = 0.10, *p* = 0.04).

**NSU:** Individuals with high levels of nomophobia significantly increase their engagement with NSU during less busy periods accompanied by negative emotions. This three-way interaction pattern is statistically significant for the frequency of NSU (*B* = 6.09, β = 0.14, *p* = 0.01). Although a similar trend for the duration of NSU was observed, the interaction is not significant (*B* = 7.75, β = 0.10, *p* = 0.09).

For the significant outcomes (e.g., SMU duration and frequency), the standardized coefficients of the focal three-way interactions ranged from 0.10 to 0.14. Though modest by conventional standards, such effect sizes are practically meaningful when accumulated across repeated daily experiences [67].

## 4. Discussion

Using intensive longitudinal and objective data, we explored the behavioral signatures of nomophobia and examined how its severity moderates the impact of daily emotional valence and situational busyness on smartphone use. Our findings indicate that behavioral differences across nomophobia levels are primarily manifested in SMU, which appears to be more sensitive than other functional categories. Furthermore, our analysis reveals a significant moderating role of nomophobia in situational behavioral patterns: individuals with higher nomophobia levels exhibited increased smartphone use—particularly SMU—in response to negative emotions during periods of low busyness.

### 4.1. Behavioral Signatures of Nomophobia

Our study offers a preliminary characterization of the behavioral signatures associated with nomophobia. The results revealed that individuals with high nomophobia exhibited significantly greater overall smartphone use and SMU compared to their low-nomophobia counterparts; however, usage patterns for IM and NSU remained statistically comparable between groups. This divergence aligns with the view that problematic smartphone use is primarily driven by passive content consumption rather than direct interactive communication [27,68]. Theoretically, these patterns may reflect the differing psychological demands inherent in each activity. IM typically necessitates active, interpersonal engagement that can be cognitively and emotionally taxing [26,32,33]. In contrast, the algorithmically curated nature of SMU facilitates “micro escape” and immediate mood regulation with minimal social or cognitive friction [30,34].

For NSU, the broad and heterogeneous nature of the category—encompassing utilities, productivity tools, and hedonic entertainment—precludes definitive interpretive claims. It is plausible that different sub-activities within NSU relate to nomophobia in opposing directions, potentially neutralizing an aggregate effect [48,69]. However, the lack of significance may stem from insufficient statistical power at Level 2 to detect smaller effect sizes, the non-significant findings warrant a nuanced interpretation.

### 4.2. Nomophobia and Coping Patterns

Building on the I-PACE model, we focused on smartphones as potential coping tools and explored how nomophobia is associated with distinct smartphone use patterns in various situations. Our multilevel regression analysis supports the hypothesis, indicating that nomophobia (Level-2 characteristic) significantly moderates the within-person (Level-1) association emotional states and levels of busyness on smartphone use. This pattern is observed across most categories (ALL, SMU, NSU), with individuals exhibiting higher levels of nomophobia being more likely to increase smartphone use frequency under negative emotions, particularly in less busy contexts. While our study does not directly measure coping styles, the behavioral proxy reveals a distinct contrast: whereas individuals with low nomophobia maintain relatively stable smartphone use regardless of emotional fluctuations, those with high nomophobia exhibit increases in usage. This suggests that they may rely heavily on their smartphones to manage negative emotions [42]. Furthermore, the observed increase in smartphone use among those with high nomophobia during less busy periods points to a pattern of using smartphone to alleviate boredom [70]. Such behavior aligns with boredom proneness, characterized by impaired impulse control and a lack of attention when bored [71,72].

The relationship between nomophobia and coping patterns can be further elucidated by examining specific smartphone use types. Our results indicate that individuals with high (versus low) levels of nomophobia exhibit significantly greater frequency and duration of SMU across various contexts. For IM, all nomophobia level groups show an increased duration of use amidst worsening emotions. This specific contrast might indicate that while individuals with nomophobia also utilize IM for coping (e.g., seeking social support or problem-solving solutions), they exhibit a disproportionate reliance on SMU for mental disengagement. Previous research corroborates that only mental disengagement, not problem-focused or socio-emotional coping, is linked to longer smartphone use durations [2].

Wolfers and Schneider [73] caution against categorizing media use as uniformly adaptive or maladaptive for coping. In alignment with this perspective, our research indicates that individuals with lower levels of nomophobia also use smartphones for coping (e.g., turning to IM). However, they do not exhibit a pronounced increase in smartphone use duration during negative emotional states or when not busy. This stability is possibly due to the availability of other coping options. Only individuals with high levels of nomophobia tend to increase their smartphone use across various coping contexts. The salience of smartphone coping may indicate a decreased ability to utilize alternative coping strategies or tools [74].

Furthermore, while we primarily interpret this smartphone use as coping, these behavioral patterns actually reflect an integration of concurrent mechanisms within the I-PACE framework [35,75]. When turning to smartphones during negative states, individuals experience not only temporary stress relief (compensation) but also concurrent digital pleasure (gratification). Through this dual reinforcement, what begins as a simple coping attempt has the potential to transition into a re-ward-driven habit. Ultimately, smartphone use may become automatic and compulsive, potentially leading to negative consequences in daily life [76].

### 4.3. Smartphone Use Indicators and Nomophobia

In our research, we examined the duration and frequency of smartphone use, discovering a strong correlation between them. This correlation is slightly higher than similar studies (e.g., Marciano and Camerini [18] reported a correlation of 0.58). This difference might be due to our focus on app session frequency, as opposed to other studies that measured smartphone pickups or unlocks. Our multilevel analysis indicates that duration and frequency exhibit a consistent relationship with nomophobia, yet they also display distinct characteristics. This distinction suggests that while these metrics are interrelated, they probably reflect unique facets of smartphone use behavior. Further exploration of the integration of both duration and frequency data could uncover deeper insights. For example, the phenomenon of “rapid checking” or “checking behavior” involves brief and repetitive smartphone interactions [77]. Analyzing these quick checks could provide a more comprehensive understanding of the compulsive checking smartphone that are symptomatic of nomophobia.

### 4.4. Implications

This study holds several implications for theory, method, and practice. Theoretically, this research extends current literature by incorporating situational variables into the investigation of nomophobia and smartphone use from a coping perspective. By applying the I-PACE model to a dynamic daily context, this study identifies situational constraints (e.g., busyness) as critical boundary conditions, which moderate the transition from internal factors (P-A-C-E) to actual behavioral execution. Additionally, the behavioral differences observed across specific smartphone use categories support the I-PACE model’s assertion regarding the necessity of differentiating specific forms of Internet use.

Methodologically, by employing an intensive longitudinal design that combines objective smartphone use tracking with daily surveys, this research advances the methodological transition in problematic smartphone use studies—shifting from the evaluation of “total usage” to a “context-sensitive” approach. Moreover, measuring different use types separately prevents the data oversimplification inherent in treating smartphone use as a uniform construct, thereby enabling a precise capture of the heterogeneous nature of specific digital behaviors.

Practically, our findings point toward potential future directions for “digital detox” interventions [78]. Specifically, our data suggest that clinical assessments of nomophobia should move beyond merely measuring total screen time and instead account for context-specific use patterns. Consequently, future interventions could explore a shift from device restriction to the development of coping skills, particularly during unstructured free time or states of negative affect. Furthermore, as for digital health systems, screen-time management tools could be optimized by replacing static restrictions with context-aware, dynamic intervention strategies.

### 4.5. Limitations and Future Study

First, as indicated by our sensitivity analyses, this Level-2 sample size limited our statistical power to detect pure between-person differences and precluded the estimation of random slopes to ensure model convergence. Consequently, the between-person comparisons should be interpreted as preliminary rather than definitive. In addition, we acknowledge that constraining random slopes may inflate the statistical significance of these cross-level interactions [61]. Future research should employ larger sample sizes to replicate and further validate these findings.

Second, our sample was restricted to Android users in East China, which may introduce systematic demographic and cultural biases. Existing evidence suggests that Android and iOS users may differ in socioeconomic status and personality traits [79,80]. Additionally, the China-specific digital ecosystem may elicit distinct usage intensities compared to Western platforms. While we categorized apps by primary function, the hybrid nature of modern applications suggests that future studies should utilize session-level feature analysis to more precisely delineate how specific sub-functions relate to nomophobic triggers.

Third, our measurement strategy prioritized ecological validity over psychometric precision. We assessed daily states using single-item scales to maximize participant compliance. However, single-item measures preclude internal consistency estimates and may introduce measurement error. Furthermore, although smartphone use tracking provides objective behavioral data, it serves only as a proxy for coping rather than a direct measure of psychological intent. However, it should be reiterated that objectively tracked smartphone use serves as a behavioral proxy for these underlying coping processes, rather than a direct psychological measure. Future studies could incorporate ultra-brief report or “think-aloud” protocols to further validate the subjective motivations behind observed behavioral reactivity.

Finally, we categorized nomophobia into “high” and “low” levels. Although this categorical approach effectively delineates distinct risk profiles [16,81], it risks oversimplifying over simplifying the continuous nature of the construct. Future research with larger samples should consider modeling nomophobia as a continuous latent variable or employing latent profile analysis to capture more nuanced transitions from functional to problematic use.

## 5. Conclusions

While the increasing prevalence of nomophobia is widely recognized, our understanding of its behavioral dynamics remains largely limited. Previous research has predominantly relied on retrospective self-reports, often overlooking the dynamic, daily situational factors. Our study addresses these issues by combining behavioral measurements of smartphone use with daily self-reported emotions and busyness, collected over a month-long period. Our analyses provided preliminary evidence linking higher nomophobia primarily with increased social media usage. Additionally, our findings lend support to the I-PACE by highlighting situation-dependent behaviors that align with specific coping patterns. Specifically, we observed that individuals with high levels of nomophobia intensify their smartphone use when experiencing negative emotions during periods of low busyness. Ultimately, by framing smartphone usage as a behavioral proxy for coping mechanisms, these results underscore the importance of considering underlying emotion regulation processes and situational contexts to better understand and manage problematic smartphone use.

## Figures and Tables

**Table 1:** Descriptive statistics and Pearson’s correlations for the investigated variables.

Variable	*M*	*SD*	1	2	3	4	5	6	7	8	9	10
1. NMP-Q	97.5	16.3	—									
2. Avg D-ALL	313.8	136.2	0.28	—								
3. Avg F-ALL	185.6	86.2	0.23	0.79 ***	—							
4. Avg D-IM	124.4	76.7	0.14	0.73 ***	0.65 ***	—						
5. Avg F-IM	81.4	41.7	0.19	0.67 ***	0.87 ***	0.84 ***	—					
6. Avg D-SMU	52.9	51.6	0.41 *	0.47 **	0.32 *	0.07	0.13	—				
7. Avg F-SMU	21.6	22.5	0.40 *	0.45 **	0.58 ***	0.18	0.39 *	0.75 ***	—			
8. Avg D-NSU	138.9	75.9	0.08	0.72 ***	0.51 ***	0.25	0.25	0.08	0.11	—		
9. Avg F-NSU	83.3	42.9	0.07	0.69 ***	0.85 ***	0.40 *	0.57 ***	0.12	0.26	0.74 ***	—	
10. Avg EV	3.4	0.5	0.16	0.13	0.14	0.09	0.08	0.05	−0.01	0.09	0.21	—
11. Avg BN	3.5	0.7	0.01	−0.14	−0.05	0.15	0.12	−0.53 ***	−0.27	−0.04	−0.08	−0.28

Notes: *N* = 37. NMP-Q (Nomophobia Questionnaire), D-ALL (Duration for ALL), F-ALL (Frequency for ALL), D-IM (Duration for Instant Messaging), F-IM (Frequency for Instant Messaging), D-SMU (Duration for Social Media Use), F-SMU (Frequency for Social Media Use), D-NSU (Duration for Non-social Use), F-NSU (Frequency for Non-social Use), EV (Emotional Valence), BN (Busyness). * *p* < 0.05. ** *p* < 0.01. *** *p* < 0.001.

**Table 2:** Multilevel linear regression analysis on smartphone use.

Independent Variables	Dependent Variables							
	D-ALL	F-ALL	D-IM	F-IM	D-SMU	F-SMU	D-NSU	F-NSU
Gender	−11.94	−35.57	−0.15	−15.35	−11.16	−12.03	0.24	−6.22
	[−96.62–72.74]	[−92.68–21.53]	[−52.86–52.56]	[−43.86–13.16]	[−36.53–14.20]	[−26.37–2.32]	[−51.89–52.37]	[−35.69–23.24]
Age	10.14 *	2.7	3.04	0.73	3.40 *	−0.15	3.52	1.84
	[1.67–18.61]	[−3.02–8.41]	[−2.23–8.31]	[−2.12–3.58]	[0.77–6.02]	[−1.64–1.34]	[−1.69–8.74]	[−1.11–4.79]
EV	−11.93 *	−2.05	−8.76 **	−2.01	−1.87	−0.68	−2.21	0.55
	[−21.60–−2.26]	[−8.05–3.94]	[−14.39–−3.13]	[−5.35–1.32]	[−6.12–2.37]	[−2.23–0.88]	[−8.55–4.13]	[−2.77–3.87]
BN	−23.43 ***	−11.95 ***	−6.13 *	−3.98 **	−8.74 ***	−3.69 ***	−9.02 **	−4.32 **
	[−31.91–−14.94]	[−17.20–−6.69]	[−11.09–−1.18]	[−6.91–−1.05]	[−12.49–−4.98]	[−5.05–−2.34]	[−14.58–−3.46]	[−7.23–−1.41]
NL	107.83 *	54.98 †	43.71	19.76	34.62 **	17.49 *	30.25	18.34
	[23.83–191.82]	[−1.63–111.60]	[−8.56–95.98]	[−8.52–48.05]	[9.92–59.31]	[3.59–31.39]	[−21.49–81.98]	[−10.88–47.57]
EV × BN	7.07 *	3.11	1.07	0.75	1.58	1.01 †	4.52 †	1.44
	[0.17–13.97]	[−1.16–7.38]	[−2.99–5.14]	[−1.63–3.14]	[−1.47–4.62]	[−0.09–2.11]	[−0.00–9.04]	[−0.93–3.80]
EV × NL	−15	−2.29	−1.36	2.06	−2.25	−0.35	−11.53 †	−3.74
	[−34.31–4.31]	[−14.25–9.68]	[−12.59–9.87]	[−4.60–8.72]	[−10.72–6.22]	[−3.45–2.75]	[−24.19–1.12]	[−10.36–2.89]
BN × NL	−23.98 **	−12.95 *	−0.18	−3.13	−13.71 ***	−5.98 ***	−10.33 †	−3.97
	[−40.67–−7.29]	[−23.29–−2.61]	[−9.92–9.56]	[−8.89–2.63]	[−21.07–−6.35]	[−8.65–−3.32]	[−21.27–0.61]	[−9.70–1.76]
EV × BN × NL	12.92 †	9.87 *	−1.16	1.45	7.07 *	2.31 *	7.75 †	6.09 *
	[−0.91–26.75]	[1.31–18.44]	[−9.31–6.99]	[−3.34–6.23]	[0.97–13.18]	[0.10–4.51]	[−1.31–16.81]	[1.35–10.83]
ICC	0.61	0.65	0.65	0.60	0.42	0.65	0.58	0.62
R^2^ (marginal/conditional)	0.192/0.686	0.126/0.696	0.082/0.679	0.087/0.637	0.194/0.533	0.187/0.712	0.068/0.608	0.082/0.649

Notes: Observations = 837, *N* = 37. Gender: 0 = female, 1 = male, NL (Nomophobia level): −1/2 = Low nomophobia, 1/2 = High nomophobia, D-ALL (Duration for ALL), F-ALL (Frequency for ALL), D-IM (Duration for Instant Messaging), F-IM (Frequency for Instant Messaging), D-SMU (Duration for Social Media Use), F-SMU (Frequency for Social Media Use), D-NSU (Duration for Non-social Use), F-NSU (Frequency for Non-social Use), EV (Emotional Valence), BN (Busyness). † *p* < 0.1. * *p* < 0.05. ** *p* < 0.01. *** *p* < 0.001.

## Data Availability

The raw data supporting the conclusions of this article will be made available by the authors on request.

## Abbreviations

The following abbreviations are used in this manuscript:

IM	Instant Messaging
I-PACE	Interaction of Person-Affect-Cognition-Execution
NMP-Q	Nomophobia Questionnaire
NSU	Non-social Use
SMU	Social Media Use
